# The prognostic value of arachidonic acid metabolism in breast cancer by integrated bioinformatics

**DOI:** 10.1186/s12944-022-01713-y

**Published:** 2022-10-15

**Authors:** Wenge Li, Xiaoyu Guo, Changzheng Chen, Juanjuan Li

**Affiliations:** 1 Department of Breast and Thyroid Surgery, Renmin Hospital of Wuhan University, 238 Ziyang Road, Wuhan, 430060 Hubei Province People’s Republic of China; 2 Department of Oncology, Shanghai Artemed Hospital, Shanghai, People’s Republic of China; 3 Department of Ophthalmology, Renmin Hospital of Wuhan University, 238 Ziyang Road, Wuhan, 430060 Hubei Province People’s Republic of China

**Keywords:** Breast cancer, Arachidonic acid metabolism, Bioinformatics, Immune

## Abstract

**Background:**

As the second cause of cancer death in women, breast cancer has become a worldwide priority. Previous studies based on tumour cell lines demonstrated that arachidonic acid (AA) and its metabolites promote cancer development. However, recent studies based on the tumour microenvironment revealed the antitumour effect of AA metabolism. Therefore, it is essential to reevaluate and elucidate the effect of AA metabolism on breast cancer.

**Methods:**

Raw data were obtained from The Cancer Genome Atlas (TCGA), Molecular Taxonomy of Breast Cancer International Consortium (METABRIC) and Gene Expression Omnibus (GEO) databases. The AA metabolic score of each sample, enrichment of differentially expressed genes (DEGs) and immune infiltration were analysed by bioinformatics. Cox regression and least absolute shrinkage and selection operator regression were performed to establish an AA metabolism prognostic signature. An AA metabolism related nomogram for predicting the survival probability of patients was built.

**Result:**

AA metabolism was related to good prognosis in the TCGA-BRCA and METABRIC cohort. DEGs enrichment suggested that the upregulated DEGs of the high AA metabolism group were significantly enriched in immune-related pathways. The high AA metabolism group was infiltrated with more CD8^+^ T cells and activated NK cells. An AA metabolic signature (SPINK8, KLRB1, APOD and PIGR) was constructed for breast cancer prognosis.

**Conclusion:**

The study indicated that a high level of AA metabolism may be a biomarker for good prognosis in breast cancer, providing a possible explanation for the discouraging effect of cyclooxygenase inhibitors in cancer therapy. Moreover, a novel AA metabolic prognostic signature was constructed in the study, providing a novel strategy for breast cancer.

**Supplementary Information:**

The online version contains supplementary material available at 10.1186/s12944-022-01713-y.

## Introduction

As the second cause of cancer death in women, breast cancer has aroused great attention [1, 2]. With the increase in body weight and decline in the fertility rate, the incidence rates of breast cancer are gradually increasing by 0.5% per year [1, 3]. Current treatments based on the clinical subtype of breast cancer, including endocrine therapy, anti-HER2 therapeutic agents and other therapies, have greatly benefited many breast cancer patients [4, 5]. However, the complex heterogeneity of breast cancer has resulted in numerous patients responding negatively to existing therapies and developing a poor prognosis [1, 2, 4]. Therefore, it is urgent to clarify the development mechanism of various subtypes of breast cancer and develop reliable tools for the guidance of individualized treatment.

Arachidonic acid (AA) is a polyunsaturated fatty acid, that is widely present in mammalian cell membranes, and is free from cell membranes after phospholipase A2 activation by neural signals [6]. AA can be metabolized into hydroxyl-eicosapentaenoic acid, epoxy-eicosatrienoic acids, prostaglandin and other active metabolites through several pathways: cytochrome P450, lipoxygenase and cyclooxygenase (COX) pathways [6–8]. Previous studies based on tumour cell lines suggested that AA and its metabolites promote the development of tumours by regulating the processes of cellular carcinogenesis, progression and differentiation, including cellular proliferation, chemotaxis, mitosis, migration and apoptosis [6, 9, 10]. Therefore, inhibitors of AA metabolism pathways, particularly COX inhibitors, has attracted great attention as promising antitumour agents. However, the results from clinical studies showed that the effectiveness of COX inhibitors in cancer was not encouraging [11–13].

Recent studies of the tumour microenvironment found that AA played an important synergistic role in the antitumour effect. One study found that ACSL4, a key inducer of ferroptosis, enhanced the ferroptosis sensitivity of breast cancer cells in an AA-dependent manner, and overexpression of ACSL4 was positively related to the sensitivity to ferroptosis induced by RSL3 in breast cancer cells [14]. Moreover, recent research found that AA played an antitumour role in three ways: promoting tumour cell ferroptosis induced by ACSL4, elevating the antitumour CD8^+^ T-cell response and sensitizing tumour cells to checkpoint therapy [15]. Therefore, it is quite necessary to reevaluate the effect of AA metabolism on breast cancer.

This study analysed the relationship between the AA metabolism level and prognosis in breast cancer. Then, the potential functions of differentially expressed genes (DEGs) and immune infiltration were evaluated. Finally, an AA metabolic prognostic signature and predictive nomogram were built and validated. The results of the current study indicated that a high level of AA metabolism is related to a better prognosis and more active immune infiltration.

## Materials and methods

### Data processing

**Fig. 1:**
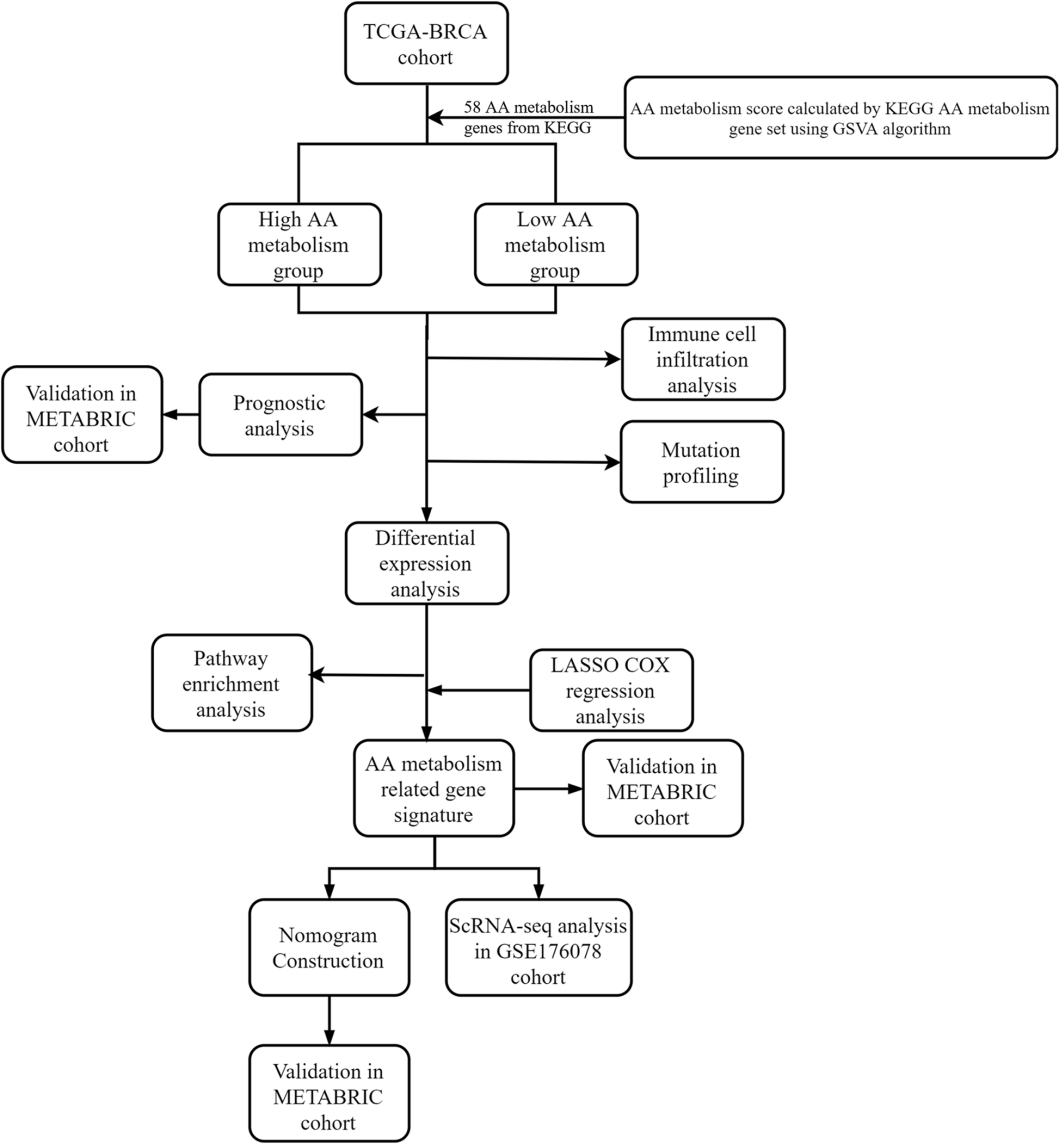
The framework of study design. TCGA, The Cancer Genome Atlas; AA, Arachidonic acid; KEGG, Kyoto Encyclopedia of Genes and Genomes; GSVA, Gene Set Variation Analysis; METABRIC, Molecular Taxonomy of Breast Cancer International Consortium; LASSO, Least absolute shrinkage and selection operator; ScRNA-seq, Single-cell RNA sequencing

The transcriptome data and clinical and mutation information were retrieved from The Cancer Genome Atlas (TCGA) (http://cancergenome.nih.gov/) and Molecular Taxonomy of Breast Cancer International Consortium (METABRIC, http://molonc.bccrc.ca/aparicio-lab/research/metabric/) databases. Information on the TCGA-BRCA and METABRIC cohorts is shown in Tables 1 and 2, respectively. The framework of study design was displayed in Fig. 1.

**Table 1:** Clinical information and its association with AA metabolism in the TCGA-BRCA cohort

Characteristics	Low (*N* = 546)	High (*N* = 545)	Total (*N* = 1091)	*P*-value
**Age**				0.20
< 60	301 (27.61%)	278 (25.50%)	579 (53.12%)	
≥ 60	245 (22.48%)	266 (24.40%)	511 (46.88%)	
**Pam50**				3.9e-25
Normal	6 (0.55%)	34 (3.12%)	40 (3.67%)	
LumA	213 (19.52%)	351 (32.17%)	564 (51.70%)	
LumB	151 (13.84%)	64 (5.87%)	215 (19.71%)	
Basal	134 (12.28%)	56 (5.13%)	190 (17.42%)	
Her2	42 (3.85%)	40 (3.67%)	82 (7.52%)	
**Stage**				0.16
I/II	407 (38.14%)	393 (36.83%)	800 (74.98%)	
III/IV	124 (11.62%)	143 (13.40%)	267 (25.02%)

**Table 2:** Clinical information and its association with AA metabolism in the METABRIC cohort

Characteristics	High (*N* = 952)	Low (*N* = 952)	Total (*N* = 1904)	*P*-value
**Age**				0.38
< 60	431 (22.64%)	411 (21.59%)	842 (44.22%)	
≥ 60	521 (27.36%)	541 (28.41%)	1062 (55.78%)	
**Pam50**				2.3e-35
Normal	49 (2.57%)	5 (0.26%)	54 (2.84%)	
LumA	400 (21.01%)	198 (10.40%)	598 (31.41%)	
LumB	287 (15.07%)	477 (25.05%)	764 (40.13%)	
Basal	90 (4.73%)	154 (8.09%)	244 (12.82%)	
Her2	126 (6.62%)	118 (6.20%)	244 (12.82%)	
**Stage**				0.98
I/II	609 (43.56%)	665 (47.57%)	1274 (91.13%)	
III/IV	60 (4.29%)	64 (4.58%)	124 (8.87%)

### Calculation of the AA metabolism-related score

Gene set variation analysis (GSVA) can estimate the activity of certain pathways based on transcriptomic data in a nonparametric and unsupervised manner [16]. GSVA was performed to enrich the AA metabolism genes and calculate the AA metabolic scores. Fifty-eight AA metabolism genes were obtained from Kyoto Encyclopedia of Genes and Genomes (KEGG) gene set collections on the Molecular Signatures Database download page (https://www.gsea-msigdb.org/gsea/downloads.jsp). The median was set as the threshold for dividing the high and low AA metabolism groups. Kaplan-Meier (KM) curves were drawn to evaluate the association of AA metabolism with prognosis.

### Screening and functional annotation of DEGs

DEGs were screened by the limma R package. *P* < 0.05 and fold change (FC) > 3/2 were defined as the selection criteria for screening the upregulated DEGs, and *P* < 0.05 and FC > 2/3 were defined as the selection criteria for screening the downregulated DEGs.

The ClusterProfiler package was used to perform Gene Ontology (GO) enrichment and KEGG enrichment. *P* < 0.05 (Fisher’s precision probability test) was considered significant.

### Evaluating immune cell infiltration

The abundance of immune cells was assessed by CIBERSORT and the relationship between the AA metabolic score and immune cell infiltration was analysed by Spearman’s correlation coefficient.

### Mutation profile analysis

The Maftools package was used to sort the mutation data. First, the mutation profiles of key genes in the AA metabolic pathways were analysed using data from the TCGA cohort. Next, the mutation profiles of the high and low AA metabolism groups were analysed.

### Development of the AA metabolism related signature (AAMRS)

The AAMRS was constructed based on the TCGA-BRCA cohort. Univariate Cox regression analysis was performed to identify genes related to overall survival (OS) from DEGs between high-and low- AA metabolism group. Next, least absolute shrinkage and selection operator (LASSO) regression was used to select candidate genes. Then, the regression coefficient and multiple regression model of genes associated with survival were determined by multivariate Cox regression. After these steps, the AAMRS was established: Risk score = Σn1 coefi*xi. The median score was set as the threshold for dividing the high and low risk groups by the AAMRS. KM curves were drawn based on the TCGA cohort for internal AAMRS validation and the METABRIC cohort for external AAMRS validation.

### Construction of the AAMRS related nomogram

Multivariate Cox analysis of AAMRS grouping and clinical factors were performed to screen independent prognostic factors. Subsequently, age, stage and AAMRS group served as parameters to construct the AAMRS related nomogram by the regplot package. Furthermore, calibration curves were applied to visualize the proximity between the predicted and factual OS. KM curves were drawn evaluate the association of AAMRS related nomogram with prognosis in the TCGA-BRCA and METEBRIC cohorts.

### AA metabolic score in the single-cell RNA sequencing (scRNA-seq) cohort

ScRNA-seq analysis was performed based on GSE176078 from the Gene Expression Omnibus (GEO) dataset ((https://www.ncbi.nlm.nih.gov/geo/). The AA metabolic score was calculated by GSVA and AAMRS distribution were visualized by the Seurat package.

### Statistical analysis

All data analyses were conducted with SPSS 22.0 or R 4.0.0. Statistical significance was determined by two-tailed t test or one-way ANOVA. The chi-square test was applied to analyse the correlation of categorical data.

## Result

### AA metabolism in breast cancer

**Fig. 2:**
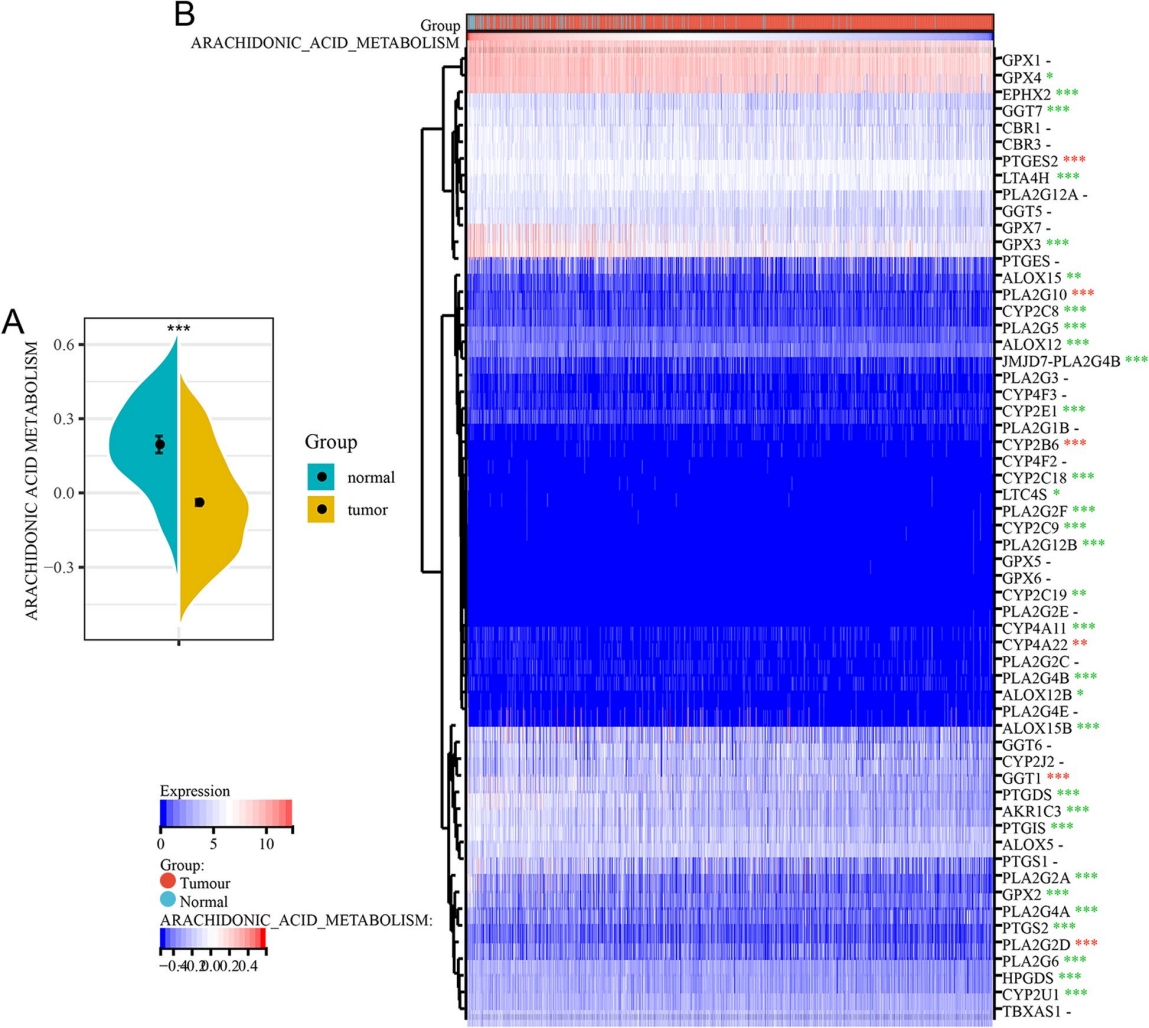
AA metabolism is more inactive in breast cancer tissues. **A** Scores of AA metabolism in normal and breast cancer tissues. **B** Heatmap of AA metabolism gene expression in normal and breast cancer tissues. * *P* < 0.05, ** *P* < 0.01, and *** *P* < 0.001

First, the AA metabolic scores of normal and breast cancer samples were compared. Analysis of the TCGA-BRCA cohort showed that the AA metabolism score of normal tissues was significantly higher than that of breast cancer tissues (Fig. 2A). The analysis of the expression of 58 AA metabolism genes expression showed that 10.3% (6/58) of genes were highly expressed in breast cancer, 36.2% (21/58) of genes had no expression difference, and 53.4% (31/58) of genes were expressed at low levels in breast cancer (Fig. 2B).

**Fig. 3:**
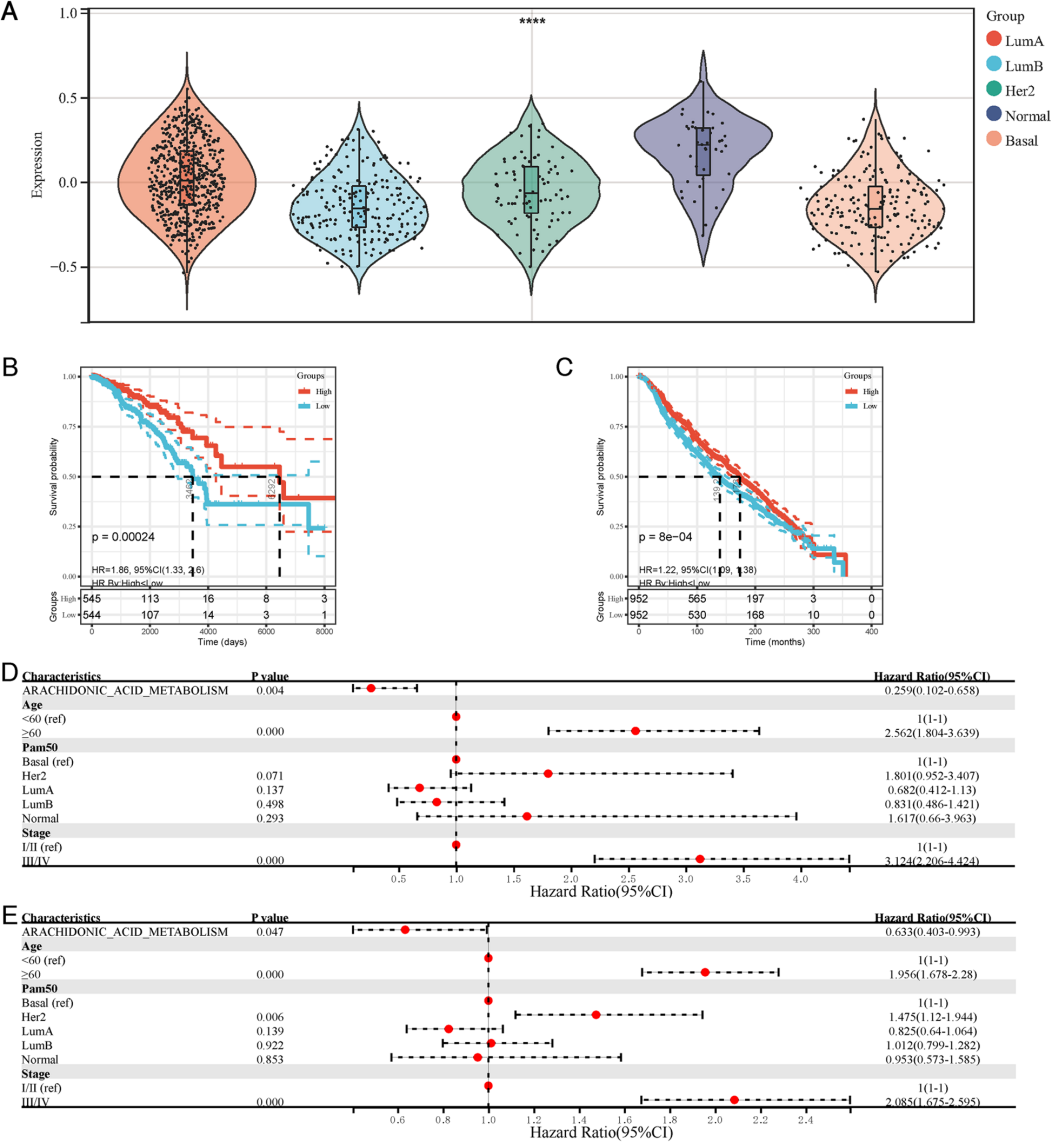
High level of AA metabolism is related to good prognosis. **A** The AA metabolic level was the highest in normal breast cancer and the lowest in basal breast cancer, **** *P* < 0.001. **B** KM curve of the AA metabolism group in the TCGA-BRCA cohort; *P* was determined by a score test using the survival package. **C** KM curve of the AA metabolism group in the METABRIC cohort; *P* was determined by a score test using the survival package. **D** The forest plot of the TCGA-BRCA cohort showed that the AA metabolism score was linked to risk. **E** The forest plot of METABRIC cohort showed that the AA metabolism score was inversely associated with risk

Then, the association between clinical factors and AA metabolism in breast cancer patients was explored (Tables 1 and 2). The AA metabolic level was closely related to the breast cancer PAM50 grouping. Moreover, AA metabolism was most active in normal breast cancer and most inactive in basal breast cancer (Fig. 3A). KM analysis showed that the high AA metabolism group had a better prognosis internally (Fig. 3B). To verify the predictive performance of AA metabolism, KM analysis in the METABRIC cohort was also performed. The OS of the high AA metabolism group was consistently higher than that of the low AA metabolism group (Fig. 3C). Considering that there was a significant difference in AA metabolism in different PAM50 genotypes, multivariate Cox regression analysis was performed, suggesting that the AA metabolic score served as an independent prognostic factor in both cohorts.

Subsequently, univariate Cox regression analysis was performed to identify survival related genes from 58 AA metabolism genes. Seven good prognostic genes and 1 poor prognostic gene were identified (Supplement Table 1). However, the good prognostic genes were not concentrated on single downstream AA metabolism pathway (Supplement Fig, 1). suggesting that the effect of AA metabolism on breast cancer maybe not realized by single AA metabolite and the underlying mechanism maybe complex.

These results indicated that a high level of AA metabolism might be a biomarker of good prognosis in breast cancer and the underlying mechanism remain to be explored.

### Identification of DEGs and functional annotations

**Fig. 4:**
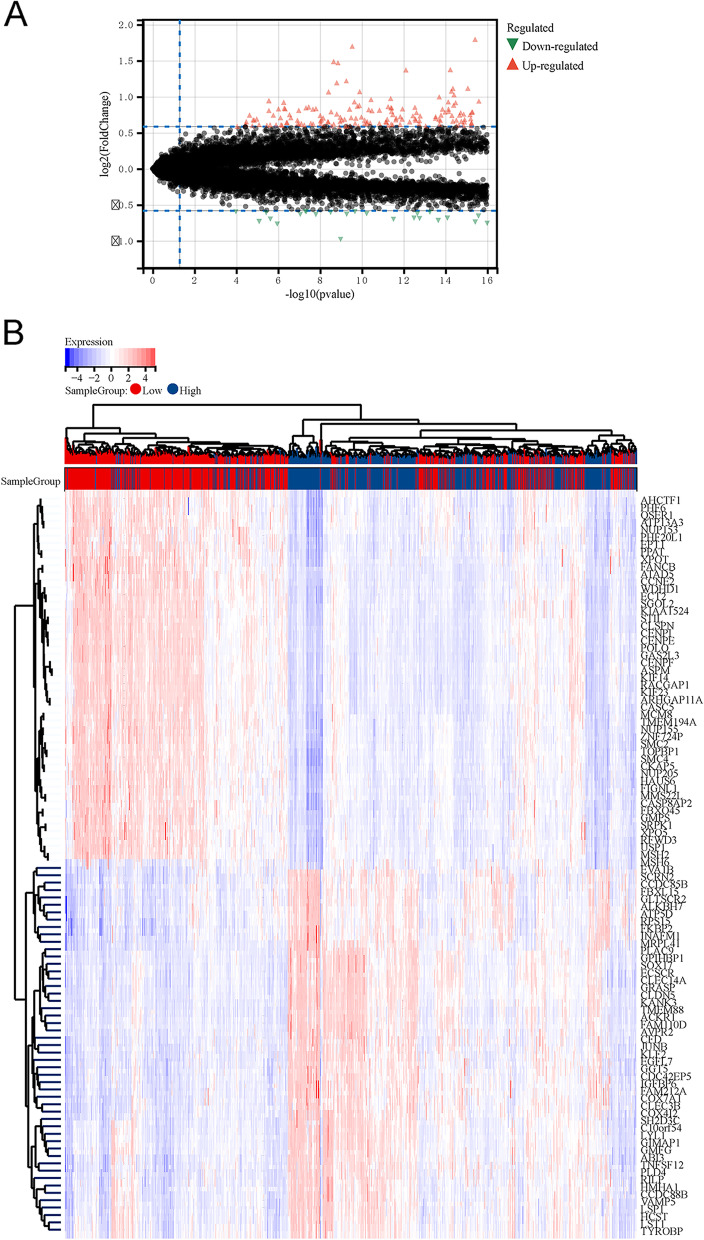
Identification of DEGs between the AA metabolism groups. **A** Volcano plot of DEGs. **B** Heatmap of DEGs; the change from red to blue represents a gradual decrease in relative gene expression

Regarding the DEGs, 437 upregulated DEGs and 398 downregulated DEGs were screened in the high AA metabolism group (Fig. 4A and B).

**Fig. 5:**
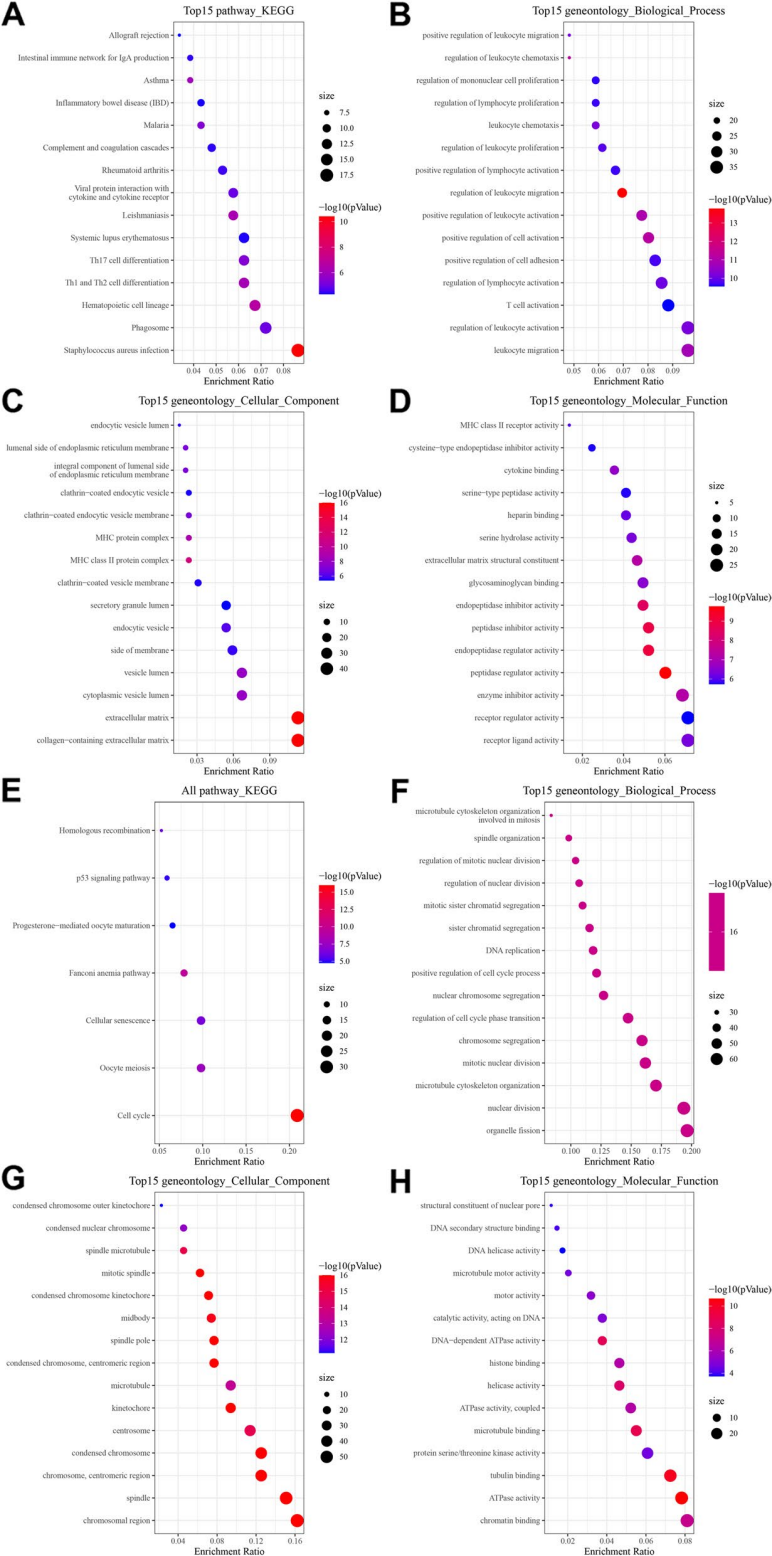
Functional annotation by KEGG and GO enrichment. **A** KEGG pathways of upregulated DEGs. **B-D** GO pathways of upregulated DEGs. **E** KEGG pathways of downregulated DEGs. **F-H** GO pathways of upregulated DEGs

Next, functional annotations of DEGs was performed. The upregulated DEGs of the high AA metabolism group were mainly enriched in immune-related pathways. The KEGG pathways were “*Staphylococcus aureus* infection”, “phagosome”, “Th1 and Th2 cell differentiation” and “Th17 cell differentiation” (Fig. 5A). GO analysis showed that the most significantly enriched pathways were “leukocyte migration” in biological process (BP), “collagen−containing extracellular matrix” in cellular component (CC), and “receptor ligand activity” in molecular function (MF) (Fig. 5B-D). For the downregulated DEGs, the functional annotations were related to cellular differentiation and progression. The KEGG pathways were “Cell cycle”, “Oocyte meiosis”, “Cellular senescence”, etc. (Fig. 5E). GO analysis showed that the most significantly enriched pathways were “organelle fission” in BP, “chromosomal region” in CC, and “chromatin binding” in MF (Fig. 5F-H).

High enrichment of immune-related pathways and low enrichment of cancer cell progression pathways may be the underlying cause of better OS in the high AA metabolism group.

### The association between AA metabolism and infiltration of immune cells

**Fig. 6:**
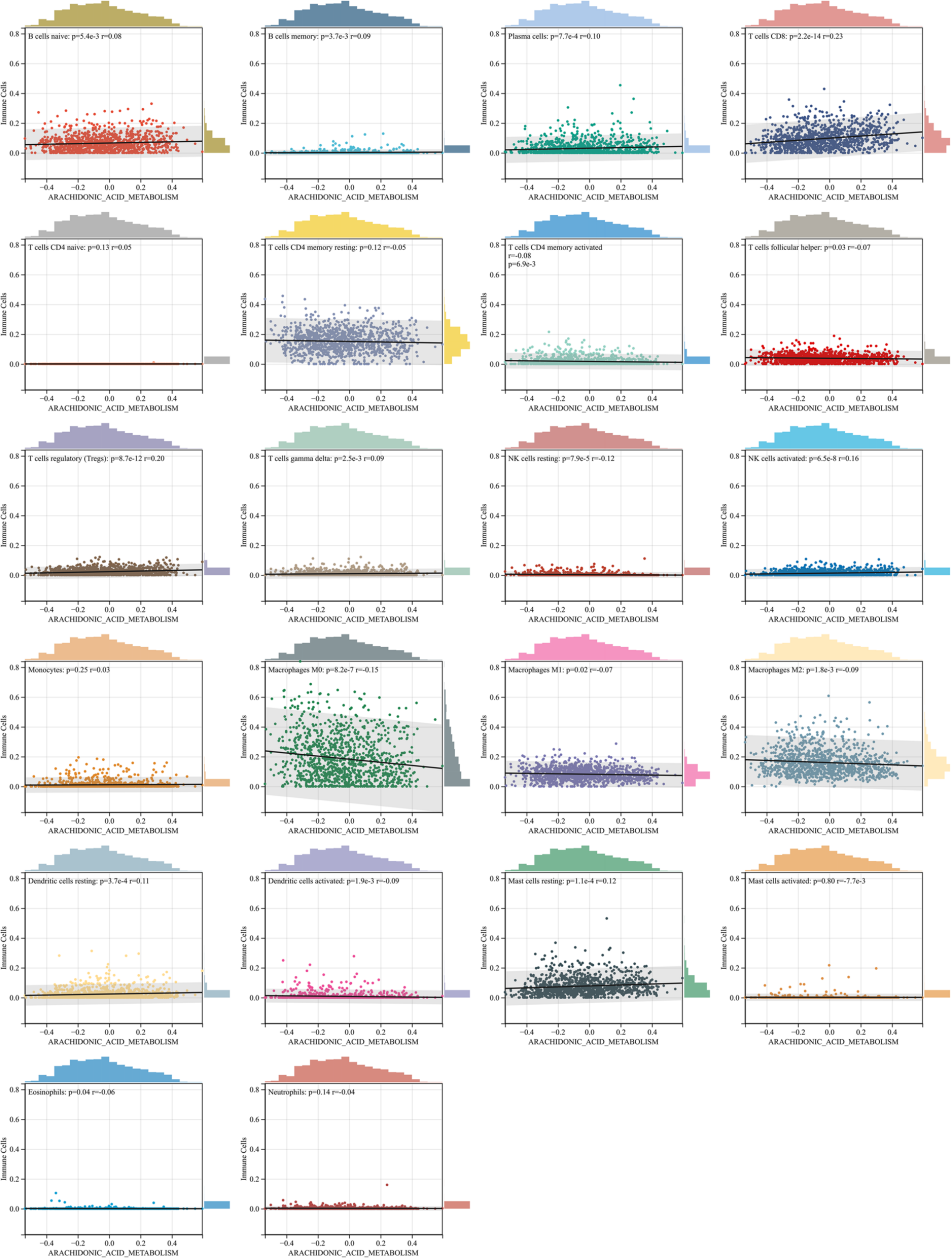
Immune cell infiltration based on the AA metabolism group. *P* < 0.05 was considered statistically significant, *R* < 0 was considered a negative correlation and *R* > 0 was considered a positive correlation

Given that the functional enrichment analysis results suggested that AA metabolism was associated with the immune response in the tumour microenvironment, the relationship between AA metabolism and the infiltration of immune cells was analysed. Scores of AA metabolism were positively correlated with the expression of plasma cells, CD8^+^ T cellss, activated NK cells, etc. Scores of AA metabolism were negatively correlated with resting NK cells, macrophages, eosinophils, etc. (Fig. 6).

### The mutation profile of AA metabolism

**Fig. 7:**
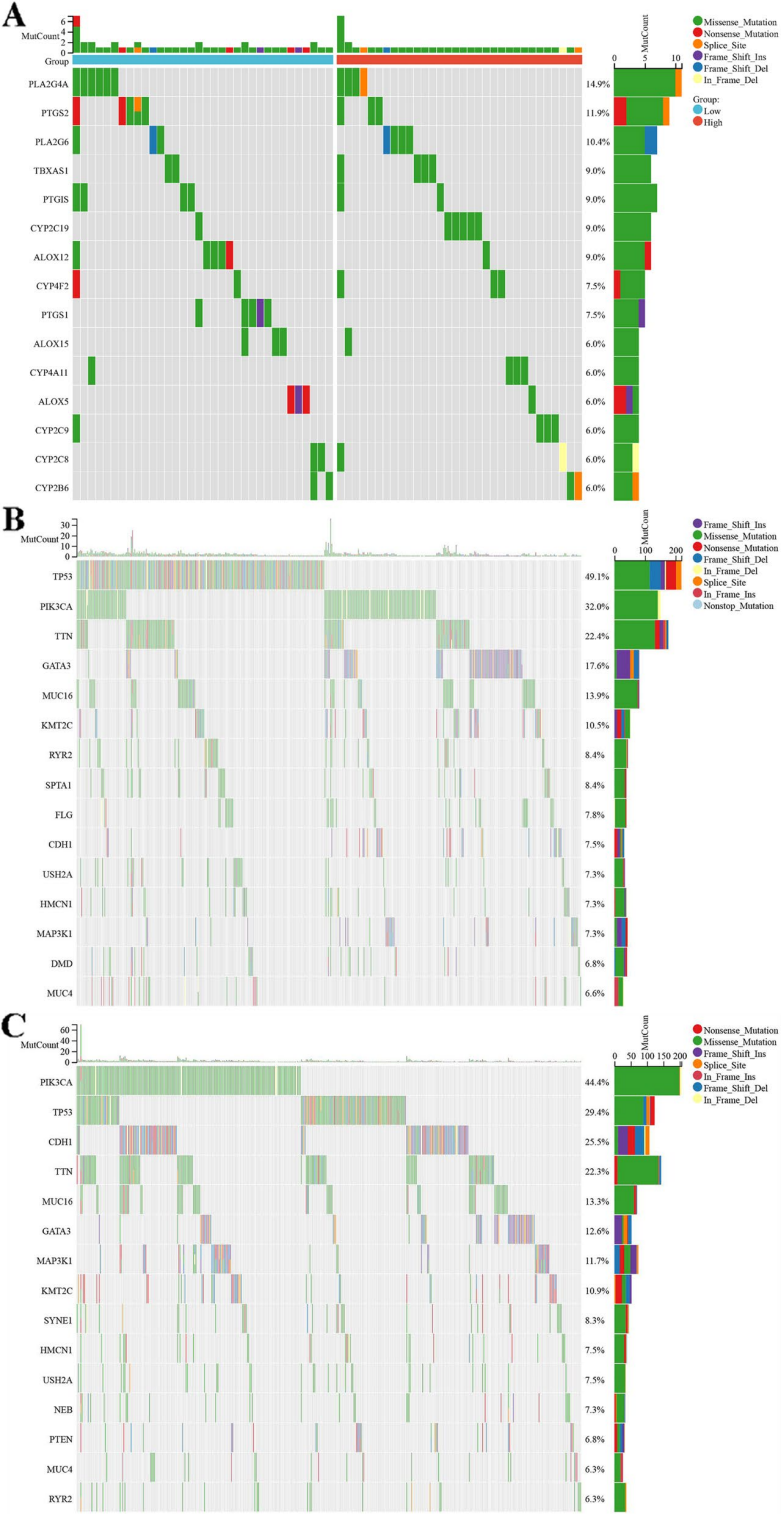
Mutation profile. **A** Mutation profile of AA metabolism genes in the TCGA-BRCA cohort: PLA2G4A was mutated most frequently (14.9%), followed by PTGS2 (11.9%) and PLA2G6 (10.4%). **B** Mutation profile of the low AA metabolism group: TP53 was mutated more frequently (49.1%). **C** Mutation profile of the high AA metabolism group: PIK3CA was mutated more frequently (44.4%)

The mutation profile related to AA metabolism was thoroughly analysed in the TCGA-BRCA cohort (Fig. 7). In the profile of key genes involved in the AA metabolism pathway, PLA2G4A was mutated most frequently (14.9%), followed by PTGS2 (11.9%), PLA2G6 (10.4%) (Fig. 7A). Then, the mutation burden of the two groups was compared. TP53 was mutated more frequently in the low AA metabolism group (Fig. 7B), while PIK3CA mutated more frequently in the high AA metabolism group (Fig. 7C).

### AAMRS construction and validation

**Fig. 8:**
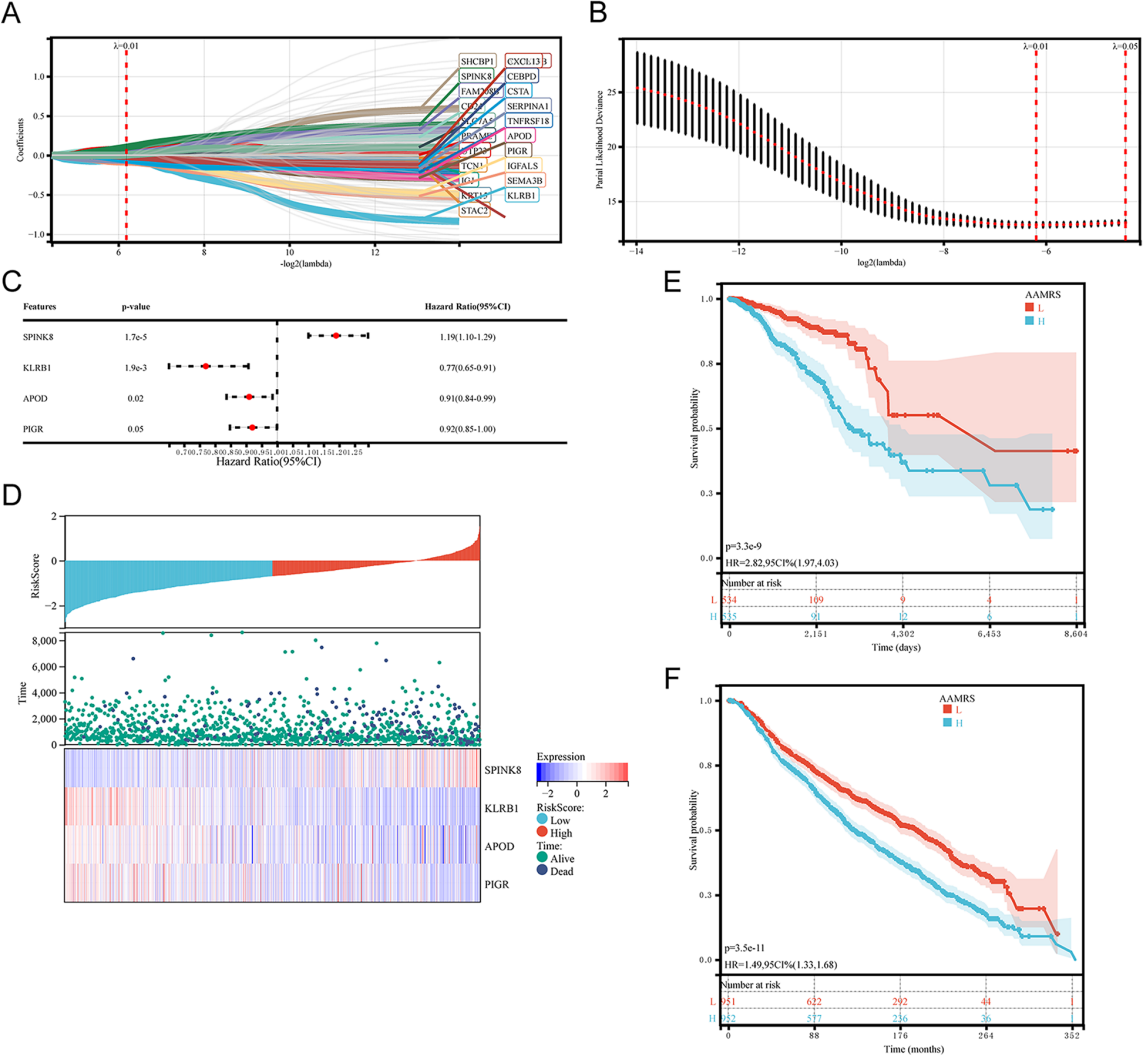
Construction and validation of the AAMRS. **A** and **B** LASSO Cox regression method (1000 iterations) was used to screen candidate genes, and the significance criterion was *P* < 0.001. **C** Multivariate Cox regression to screen key genes thoroughly. **D** Risk score and survival status in AAMRS. **E** KM curve of the AAMRS internal validation in the TCGA-BRCA cohort. **F** KM curve of the AAMRS external validation in the METRABIC cohort

To construct the AAMRS, univariate Cox regression screened 165 OS-related genes from DEGs between high- and low- AA metabolism group. To avoid overfitting the AAMRS, LASSO regression analysis (Fig. 8A, B) and multivariate Cox regression (Fig. 8C) were further performed. Finally, 4 genes were identified to establish the AAMRS: Risk score = 0.17661*SPINK8–0.26264*KLRB1–0.09641*APOD -0.0832*PIGR.

The median of AAMRS divided patients into two risk subgroups (Fig. 8D). Then, the predictive performance of the AAMRS was validated using KM survival curves. The survival probability of patients in the high AAMRS risk group was significantly poorer in the TCGA-BRCA cohort (Fig. 8E). The KM survival curves in the METABRIC cohort showed consistent results with those in the TCGA-BRCA cohort (Fig. 8F).

### Nomogram variable screening, construction and validation

**Fig. 9:**
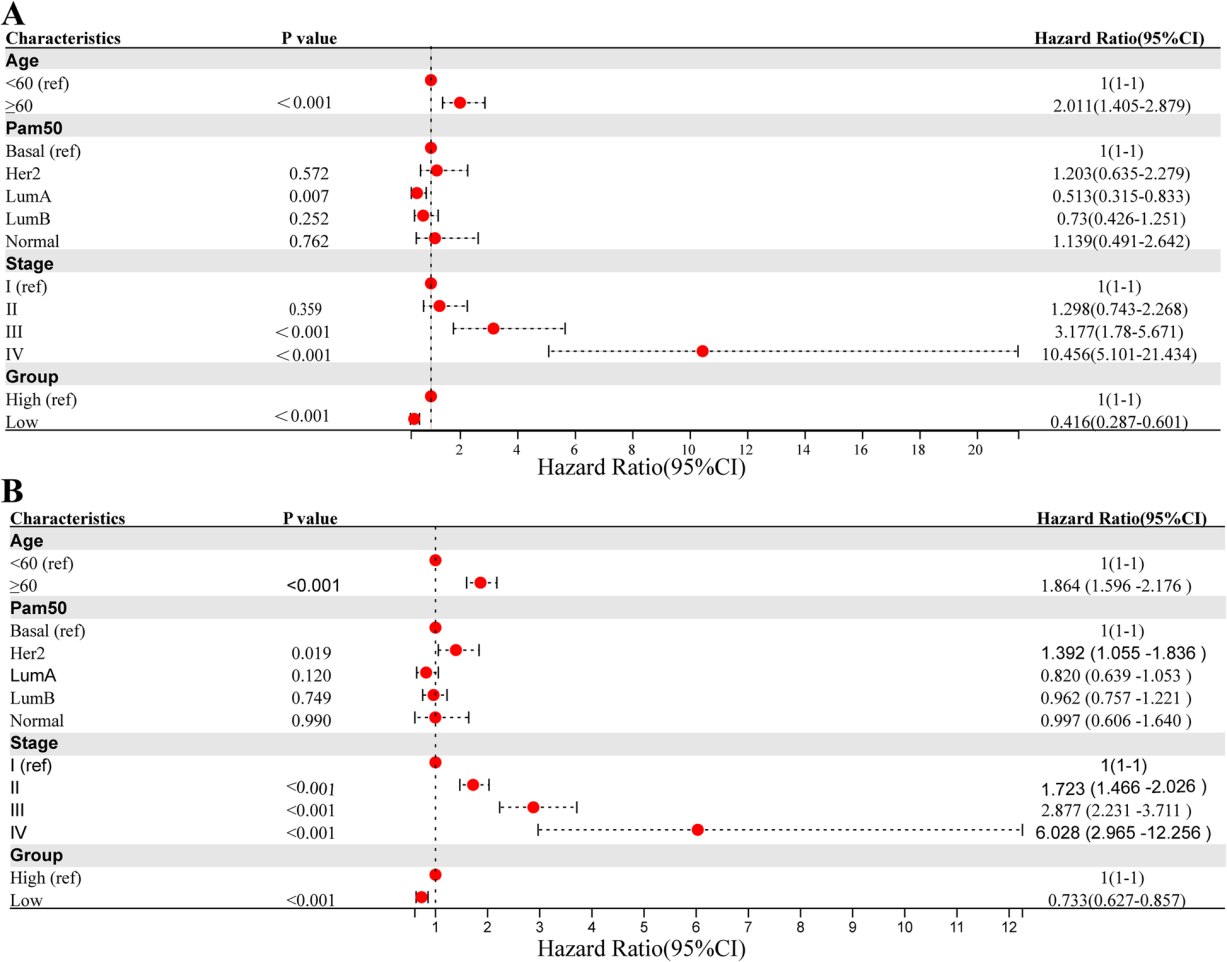
The forest plots for multivariate regression of clinical factors and AAMRS. **A** Age, AAMRS group and tumour stage were independent prognostic factors in the TCGA-BRCA cohort. **B** Age, AAMRS group and tumour stage were independent prognostic factors in the METABRIC cohort

**Fig. 10:**
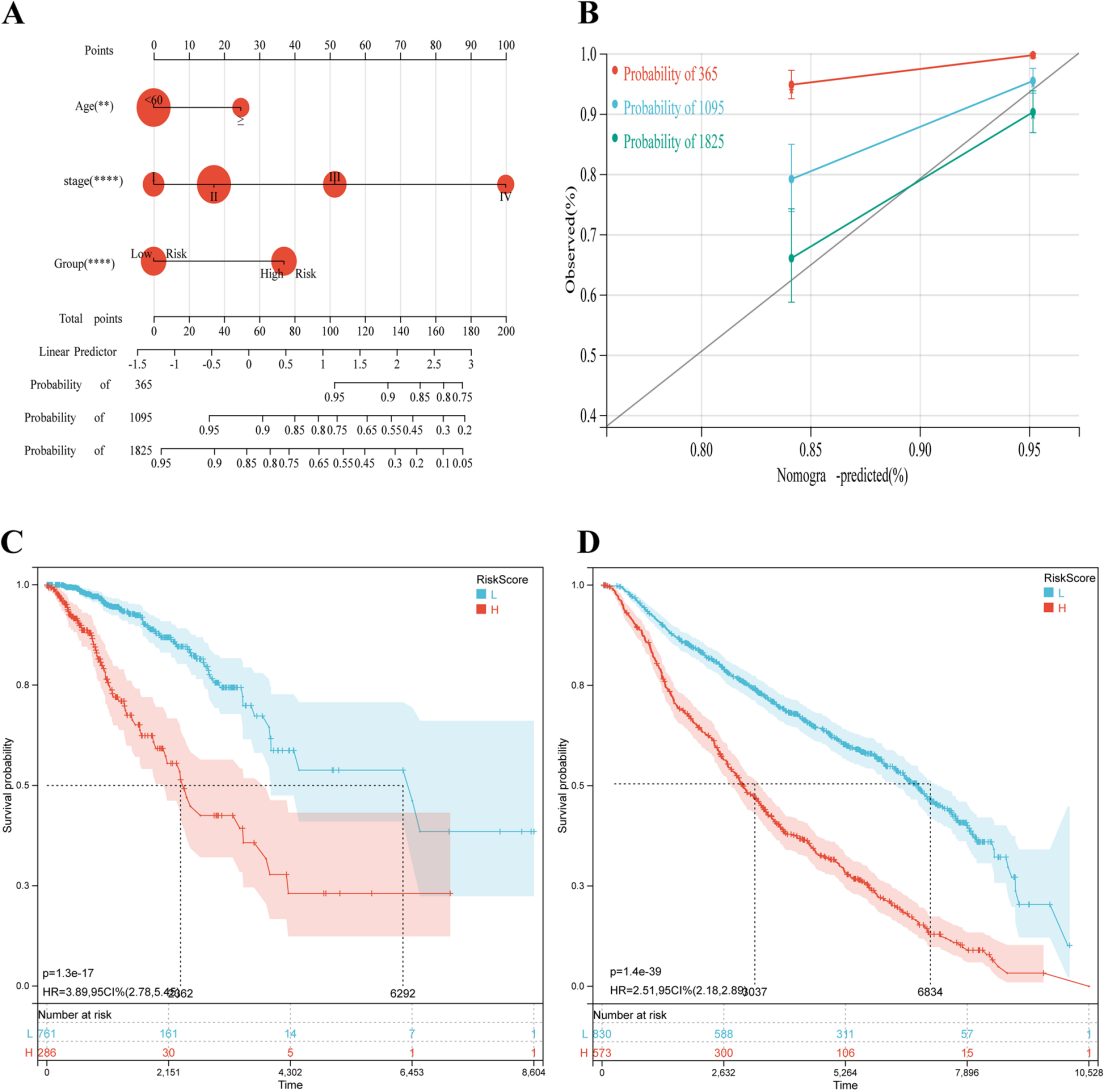
Establishment of the AAMRS related nomogram. **A** Nomogram for predicting the OS probability, by setting age, AAMRS group and tumour stage as parameters. **B** Calibration curves for verifying the prediction accuracy; red represents the 1-year prediction, blue represents the 3-year prediction, and green represents the 5-year prediction. **C** KM curve of the AAMRS related nomogram validation in the TCGA-BRCA cohort. **D** KM curve of the AAMRS related nomogram validation in the METABRIC cohort

Multivariate Cox regression was performed to select the variables for forest plot (Fig. 9A, B). According to the forest plot, age, tumour stage and AAMRS group could serve as independent prognostic factors. Then, a novel predicting nomogram was built, with age, tumour stage and AAMRS group as parameters (Fig. 10A). The calibration curves showed that the AAMRS related nomogram accurately predicted the survival probability (Fig. 10B). The KM survival curves confirmed the predictive ability of the nomogram in the TCGA-BRCA (Fig. 10C) and METABRIC cohorts (Fig. 10D).

### ScRNA-seq revealed AA metabolic characteristics and AAMRS distribution in breast cancer

**Fig. 11:**
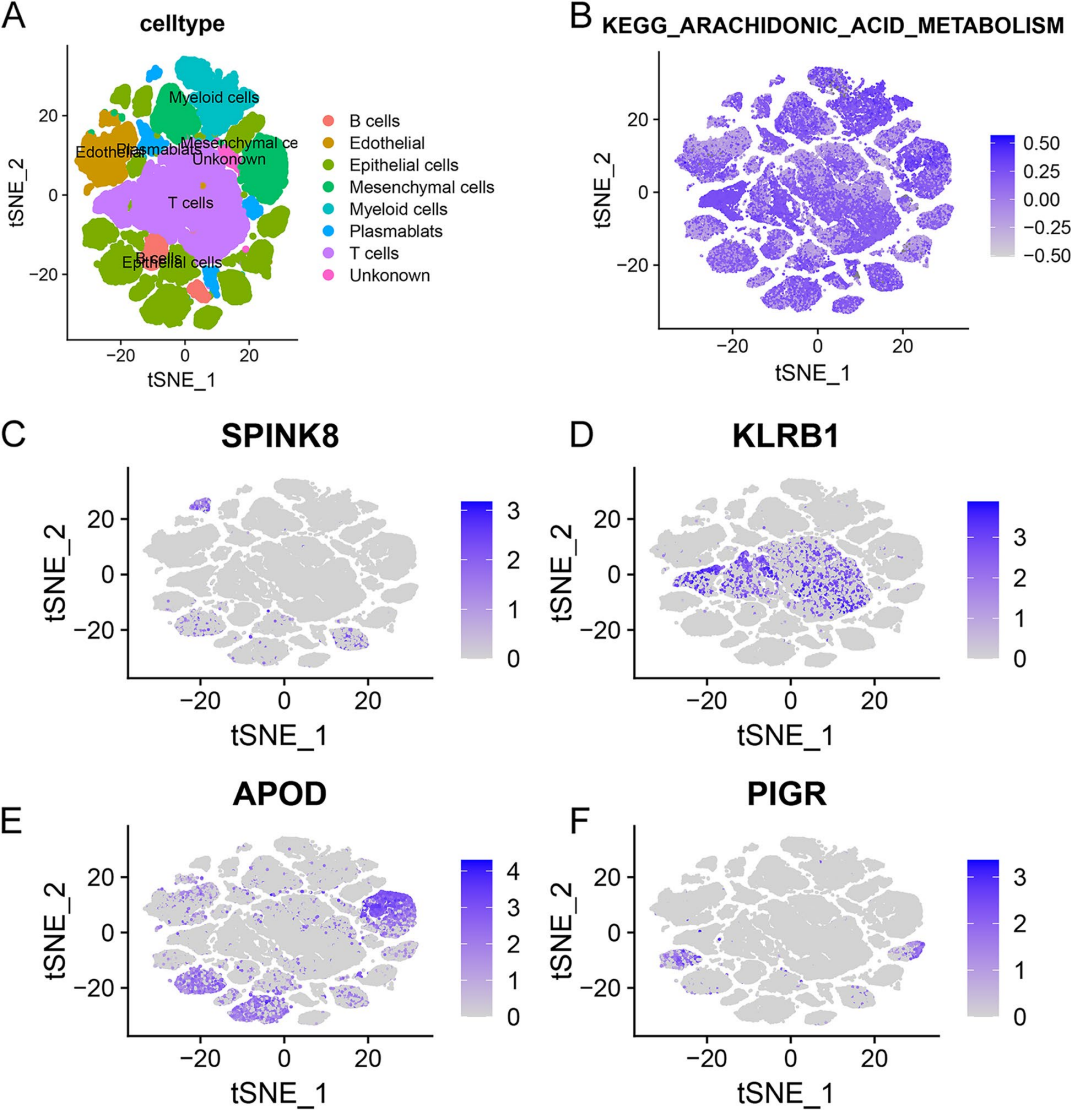
Sc-RNA-seq analysis. **A** The distribution of various cell types in breast cancer. **B** AA metabolism was widely existed in different cell types. **C-F** Expression of SPINK8, KLRB1, APOD and PIGR

The AA metabolic characteristics and prognostic gene expression characteristics of the scRNA-seq data from GSE176078 were analysed (Fig. 11A). The results showed that AA metabolism was not cell specific and widely existed in different cell types (Fig. 11B). For the AAMRS, SPINK8 and PIGR were mainly expressed in some epithelial cells, KLRB1 was widely expressed in T cells, and APOD was expressed in mesenchymal, endothelial and epithelial cells (Fig. 11C-F).

## Discussion

Breast cancer consists of different heterogeneous subtypes, and each subtype has its own biological and clinical characteristics [17]. One of the current hotspots in breast cancer is the discovery of reliable prognostic biomarkers to identify high-risk breast cancer patients who could benefit from intensive treatment [18]. The rapid progression of genome, transcriptome and bioinformatics approaches has advanced the process of cancer biomarker discovery and personalized cancer treatment over the last decade, providing an aid to treatment decisions in cancer [19–21]. By utilizing a bioinformatics approach and RNA-seq data, the present study confirmed that AA metabolism could be a potential factor for breast cancer prognosis and is related to good outcomes for breast cancer. Moreover, a reliable AA metabolic prognostic signature was built for the prognostic prediction of breast cancer, which may help to improve the diagnosis and personalized treatment of breast cancer patients.

### Comparisons with other studies and what does the current work add to the existing knowledge

AA is ubiquitously expressed in every mammalian cell membrane and participates in metabolic activities throughout the whole cell cycle, including cancel cell growth and death [22]. Previous studies of tumour cell lines have mainly focused on the effect of AA and its metabolites on cancer progression through COX and other pathways. Therefore, much more effort has been devoted over the last decade to determine whether COX inhibitors could serve as promising agents for cancer therapy. However, the results are conflicting. Some studies suggested that COX inhibitors could reduce the risk of breast, stomach and colorectal cancers [8, 23]. In contrast, no protective effect of COX inhibitors also been generally reported [12, 24, 25]. More surprisingly, some studies reported that the use of COX inhibitors was related to an increased risk of cancer incidence and mortality [11, 12]. This contradiction suggests that our understanding of AA metabolism in cancer is still limited.

Recent studies revealed the important role of AA metabolism in cancer cell death rather than cell growth. Experimental evidence based on many tumour cell lines proved that AA could induce tumour cell ferroptosis with IFN-γ and enhance antitumour immunity with CD8^+^ T cell [15]. More importantly, AA was essential to enhance the ferroptosis sensitivity induced by ACSL4 in breast cancer [14]. Moreover, the increased level of AA promoted the sensitivity of gastric tumours to ferroptosis, and supplementation with AA was essential to induce gastric tumours to ferroptosis [26]. Consistent with these findings, this study found that the infiltration levels of CD8^+^ T cells were higher in high AA metabolism group. The process by which CD8^+^ T cell suppress tumour development by inducing ferroptosis is a recently reported novel mechanism, and AA plays a synergistic role in this process [15, 27, 28]. A high level of AA could increase the function of CD8^+^ T cells in tumour cell ferroptosis. Moreover, the therapeutic efficacy of checkpoint blockade could also be sensitized by AA [15]. In the present study, KEGG showed that the upregulated genes of high AA metabolic group were mainly associated with immune related pathways, suggesting that the immune infiltration of the high AA metabolic group is more active and intense in the tumour microenvironment. Therefore, a high level of AA metabolism may improve the prognosis of breast cancer by enhancing the induction of CD8^+^ T cell on tumour cell ferroptosis and promoting immune responses to increase the sensitivity to checkpoint blockade therapy in tumour microenvironment.

Chronic inflammation is closely linked to the occurrence of cancer [29]. Overexpression of AA and its metabolites promotes the process of chronic inflammation and precancerous lesions. Numerous studies have proven that early intervention of COX inhibitors reduces the risk of cancer occurrence [8, 23]. However, once the tumour occurrs and advances, AA and its metabolites in the tumour microenvironment play a protective role against the tumour development [30]. This study found that a high level of AA metabolism in breast cancer is related to more active immune responses, which provides a reasonable explanation for the contradictory effect of COX inhibitors and helps to better clarify the role of AA metabolism in cancer.

In this study, four genes were identified in the AAMRS. KLRB1, APOD and PIGR were associated with low risk, while SPINK8 was associated with high risk. KLRB1 encodes CD161, which is expressed on many T-cell subtypes. A previous breast cancer signature included this gene as a biomarker for good prognosis [31, 32]. A previous pan-cancer study also confirmed that upregulation of KLRB1 was related to good prognosis in most cancers, including breast cancer [33]. Experimental evidence proved that CD8^+^ CD161^+^ T cells exerted cytotoxicity against tumour cells and protected mice from tumours, while CD8^+^ CD161^−^ T cells could not [34, 35]. This study found that KLRB1 was widely expressed in T cells, consistent with the conclusion that high expression of KLRB1 in T cell indicating a better prognosis. APOD encodes a component of high density lipoprotein involved in lipid metabolism and neuroprotection. This study found that high APOD expression is related to longer survival, which is consistent with a previous breast cancer signature [36]. One study pointed out that APOD was most highly expressed in benign tumours and least expressed in invasive cancer and breast cancer with metastasis [37]. Interestingly, APOD could bind to AA and change the end products of AA metabolism to reduce the activity of tumour cells [37]. Therefore, the alternation of AA metabolism may be a contributor as well as a result of altered APOD expression and the underlying mechanism needs deeper investigation. PIGR encodes the partial immunoglobulin molecules of IgA and IgM. Although PIGR has been proven to promote hepatocellular carcinoma aggressiveness, the role of PIGR in breast cancer is not clear [38]. A gene signature of luminal breast cancer indicated that PIGR served as one biomarker for good outcome [39]. Moreover, the PIGR expression is downregulated in breast cancer tissues compared with paracancerous tissues [40]. This study also suggests that high PIGR expression is a biomarker for better OS and Its role in breast cancer progression appears to be entirely different from that of liver cancer, while the conclusion should be supported by more evidence. Unlike the roles of KLRB1, APOD and PIGR, the role of SPINK8 has not been reported previously. To the best of our knowledge, this the first study reported the expression of SPINK8 in the tumour cells. This study first identified SPINK8 as a biomarker for poor prognosis in breast cancer, and the role of SPINK8 should be fully elucidated.

### Study strengths and limitations

The greatest strength of this study is the assessment of the role of AA metabolism in breast cancer from the perspective of the tumour microenvironment as a whole by bioinformatics, thus overcoming the limitation of previous studies solely focused on tumour cell lines. However, the study was retrospective because raw data were obtained from public datasets. Furthermore, more in vitro or in vivo evidence is needed to validate the results. Finally, the underlying mechanisms of the four independent prognostic genes were not further explored. Therefore, functional experiments of the four independent prognostic genes were needed to validate the fundings and clarify the potential molecular mechanism involved.

## Conclusion

The present study indicated that a high level of AA metabolism might be a biomarker of good prognosis in breast cancer and developed a novel AAMRS for breast cancer. Moreover, a high level of AA metabolism was closely linked to immune infiltration, providing a possible explanation for the discouraging effect of COX inhibitors in cancer therapy. Therefore, this study suggests that the role of COX inhibitors in cancer treatment should be cautiously reviewed.

## Supplementary Information

**Additional file 1: Supplement Table 1.** Survival related AA metabolism genes.

**Additional file 2: Supplement Fig. 1.** Distribution of survival related AA metabolism genes in AA metabolism network.

## Data Availability

The raw data of our study were downloaded from TCGA dataset (http://cancergenome.nih.gov/), METABRIC dataset (http://molonc.bccrc.ca/aparicio-lab/research/metabric/) and GEO dataset (https://portal.gdc.cancer.gov/).

## Abbreviations

AA: Arachidonic acid
COX: Cyclooxygenase
TCGA: The Cancer Genome Atlas
METABRIC: Molecular Taxonomy of Breast Cancer International Consortium
GEO: Gene Expression Omnibus
GSVA: Gene Set Variation Analysis
KEGG: Kyoto Encyclopedia of Genes and Genomes
KM: Kaplan-Meier
DEGs: Differentially expressed genes
FC: Fold change
GO: Gene Ontology
AAMRS: Arachidonic acid metabolism related signature
OS: Over survival
LASSO: Least absolute shrinkage and selection operator
ScRNA-seq: Single-cell RNA sequencing
BP: Biological process
CC: Cellular component
MF: Molecular function
