# Supplementary material for: The prognostic value of arachidonic acid metabolism in breast cancer by integrated bioinformatics
Source: Lipids Health Dis. 2022 Oct 15;21:103. doi: 10.1186/s12944-022-01713-y (PMC9569099; doi:10.1186/s12944-022-01713-y)
Supplement: Supplementary file 1 — Additional file 1: Supplement Table 1. Survival related AA metabolism genes. [file 12944_2022_1713_MOESM1_ESM.docx]

**Supplement table 1** Survival related AA metabolism genes

| Genes | HR | Lower | Upper | p |
| --- | --- | --- | --- | --- |
| PLA2G1B | 0.468534 | 0.311894 | 0.703842 | 0.000243 |
| ALOX15 | 1.200247 | 1.07009 | 1.346236 | 0.001718 |
| PLA2G2D | 0.849595 | 0.762258 | 0.94694 | 0.003003 |
| CBR1 | 0.813939 | 0.701492 | 0.94441 | 0.006678 |
| LTA4H | 0.724646 | 0.537854 | 0.976311 | 0.034572 |
| ALOX15B | 0.921709 | 0.850882 | 0.99843 | 0.045025 |
| CYP4F3 | 0.804946 | 0.648653 | 0.998898 | 0.047665 |
