# Supplementary figures and images for: The prognostic value of arachidonic acid metabolism in breast cancer by integrated bioinformatics
Source: Lipids Health Dis. 2022 Oct 15;21:103. doi: 10.1186/s12944-022-01713-y (PMC9569099; doi:10.1186/s12944-022-01713-y)

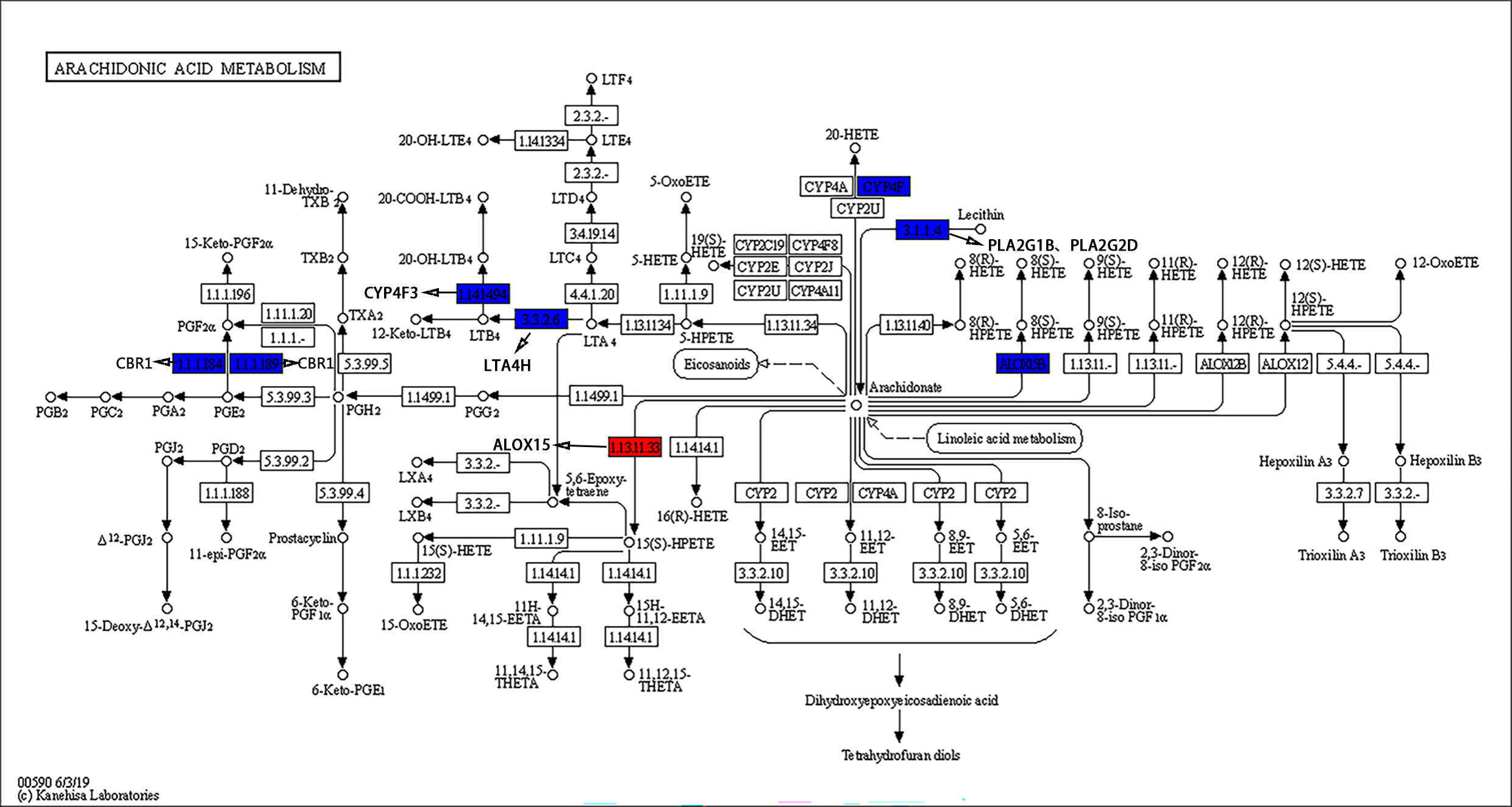

Supplement: Supplementary file 2 — Additional file 2: Supplement Fig. 1. Distribution of survival related AA metabolism genes in AA metabolism network. [file 12944_2022_1713_MOESM2_ESM.tif]
